# Association between dietary inflammatory index and chronic kidney disease in middle-aged and elderly populations

**DOI:** 10.3389/fnut.2024.1335074

**Published:** 2024-01-17

**Authors:** Meiqian Guo, Yi Lei, Xueqing Liu, Xiang Li, Yong Xu, Donghui Zheng

**Affiliations:** ^1^Department of Nephrology, The Affiliated Huai’an Hospital of Xuzhou Medical University and Huai’an Second People’s Hospital, Huai’an, China; ^2^Key Laboratory for Chronic Kidney Disease of Xuzhou Medical University, Xuzhou Medical University, Huai’an, China; ^3^Huai’an Key Laboratory of Chronic Kidney Disease, The Affiliated Huai’an Hospital of Xuzhou Medical University and Huai’an Second People’s Hospital, Huai’an, China

**Keywords:** dietary inflammatory index, NHANES, population aging, estimated glomerular filtration rate, albuminuria, chronic kidney disease

## Abstract

**Background:**

A link between food-induced inflammation and common chronic diseases has been identified in studies. However, there was uncertainty about the influence of dietary inflammatory potential on the risk of chronic kidney disease (CKD) among middle-aged and older groups. Our research aimed to examine the connection between dietary inflammatory index (DII) to CKD in people aged 40 years and older.

**Methods:**

This study comprised ten cycles of the National Health and Nutrition Examination Survey (NHANES) from 1999 to 2018. Linear associations of DII with CKD, low-eGFR, and albuminuria were examined using multiple logistic regression, whereas non-linear associations were assessed by smoothed curve fitting. Besides, we conducted subgroup analyses and interaction tests.

**Results:**

Of the 23,175 middle-aged and older individuals, a total of 5,847 suffered from CKD, making up 25.23% of all participants. After adjustment for all covariates, we found that increased DII scores were positive with an increased hazard of CKD (OR = 1.08, 95% CI: 1.05, 1.10, *p* < 0.0001), and the same was shown between DII and low-eGFR (OR = 1.16, 95% CI: 1.13, 1.19, *p* < 0.0001). After further converting DII into categorical variables, the above relationship still existed. These relations were consistent in different ages, genders, BMI, whether smoking, whether suffering from hypertension, and whether suffering from diabetes, with no significant stratification differences (all P for interaction >0.05). Surprisingly, we did not find a statistically significant correlation of DII to albuminuria after complete adjustment for covariates (OR = 1.02, 95% CI: 1.00, 1.05, *p* = 0.0742). Even when DII was considered as a categorical variable, this relation was still not statistically significant. Furthermore, we found an association in the shape of a U between DII and low-eGFR in the fully adjusted model, with a turning point at a DII of 1.6.

**Conclusion:**

Our findings indicated that middle-aged and older persons with greater levels of DII had a significantly higher risk of CKD.

## 1 Introduction

Chronic kidney disease (CKD) is known to be a common chronic disease, especially in the elderly population (1). Its most prominent feature is the damage to kidney structure and function caused by a variety of reasons (2). It has been shown that between 1990 and 2016, the global burden of CKD rose by 87%, and that CKD fatalities have climbed by 100%, becoming one of the top non-communicable causes of mortality globally (3–5). A growing number of older persons are suffering from CKD as the population ages (6). CKD is closely linked to appetite loss, falls and fractures, cognitive decline, diminished physical function, poor quality of life, and inflammation. In recent years, it has also steadily gained recognition as a factor in hastened aging (7, 8). For the prevention and treatment of CKD as well as to relieve the global healthcare burden, it is crucial to actively explore the causes underlying CKD in middle-aged and elder individuals.

Diet plays a central role in regulating inflammatory processes and is closely related to the occurrence and progression of various chronic diseases. The Dietary Inflammatory Index (DII) is an indicator that has been widely used in recent years for the assessment of the inflammatory potential of individual diets (9). The DII scoring system consists of 45 food nutrients. Each food parameter was scored according to its effect on pro-inflammatory markers interleukin-1β (IL-1β), IL-6, tumor necrosis factor α (TNFα), C-reactive protein (CRP) and anti-inflammatory markers (IL-4, IL-10): “-1” indicates an anti-inflammatory effect, “ + 1” indicates a pro-inflammatory effect and “0” indicates that the food parameter had no effect on inflammatory markers. After a series of calculations and weighting, a complete evaluation system for dietary inflammation was ultimately developed (10). Most importantly, the DII assesses the combined effects of multiple dietary components, not just one nutrient or food. Positive DII scores indicate pro-inflammatory diets, while negative DII scores indicate anti-inflammatory diets, with higher absolute values indicating greater anti-inflammatory/pro-inflammatory effects. It has been proposed that numerous inflammatory disorders, including obesity, diabetes, and cardiovascular disease, have a favorable correlation with DII scores (11–15). Systemic low-grade inflammation is one of the most important pathogenic processes in CKD, and it is also influenced by the dietary status of patients. However, there remained a dearth of relevant research on the connection of DII to CKD in the middle-aged and elderly population.

Therefore, we conducted this study to look into the connection of DII to CKD in people aged 40 years and older.

## 2 Materials and methods

### 2.1 Study population

Data for our study came from the 1999 to 2018 National Health and Nutrition Examination Survey (NHANES). NHANES is a research project hosted by the National Centre for Health Statistics (NCHS). Updating of its data is still in progress. The sample utilized in this investigation, which employed stratified multistage random sampling, was well-represented. The study had the approval of the NCHS Ethical Review Committee, and written informed consent was obtained from all subjects. All information from our research is available on the NHANES website.^1^

**FIGURE 1:**
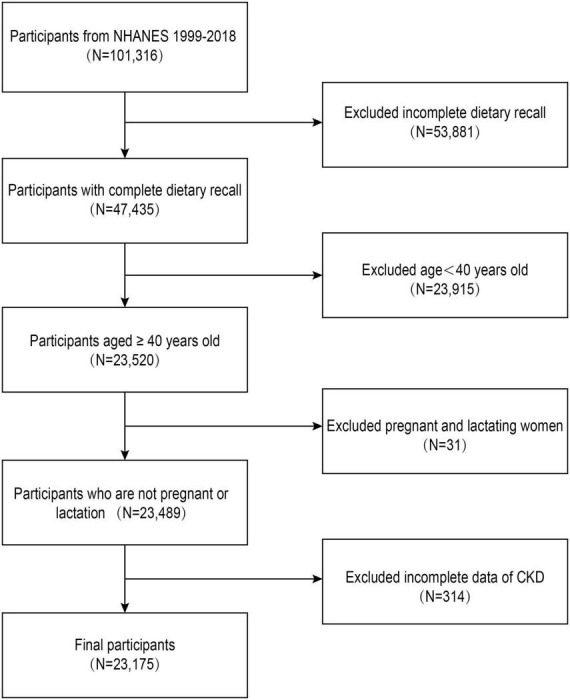
Flowchart of the sample selection.

Initially, there were 101,316 participants. After excluding those with incomplete or missing dietary information (*n* = 53,881), those under the age of 40 (*n* = 23,915), those who were pregnant or breastfeeding (*n* = 31), and those with missing information on CKD (*n* = 314), our final analysis comprised 23,175 participants (Figure 1).

**TABLE 1:** Baseline characteristics of study participants.

	Tertile 1 (−5.28–0.75)	Tertile 2 (0.76–2.47)	Tertile 3 (2.48–5.79)	*P*-value
	*N* = 7,725	*N* = 7,725	*N* = 7,725	
Age (year), (median, IQR)	59.00 (49.00–70.00)	60.00 (49.00–70.00)	61.00 (50.00–71.00)	<0.001
**Sex (%)**				0.039
Male	50.60	48.66	50.19	
Female	49.40	51.34	49.81	
**Race (%)**				<0.001
Mexican American	5.84	5.86	5.66	
Other Hispanic	3.97	4.60	4.74	
Non-Hispanic White	77.64	74.82	72.26	
Non-Hispanic Black	6.49	9.48	12.38	
Other Race	6.05	5.24	4.96	
**Education level (%)**				<0.001
Less than high school	4.80	7.40	8.74	
High school or GED	29.42	36.12	43.48	
Above high school	65.73	56.44	47.68	
Unknown	0.06	0.05	0.10	
**Smoking status (%)**				<0.001
Never	51.62	51.15	46.93	
Former	35.22	29.81	29.06	
Current	13.09	19.00	23.97	
Unknown	0.07	0.04	0.04	
PIR, (median, IQR)	2.91 (1.47–5.00)	2.31 (1.24–4.31)	1.92 (1.10–3.57)	<0.001
Serum iron (mg/dl), (median, IQR)	84.00 (65.00–106.00)	81.00 (62.00–104.00)	78.00 (59.00–100.00)	<0.001
Potassium (mmol/L), (median, IQR)	4.10 (3.85–4.30)	4.00 (3.80–4.28)	4.00 (3.80–4.20)	<0.001
Albumin (g/dl), (median, IQR)	4.30 (4.10–4.50)	4.20 (4.00–4.40)	4.10 (3.90–4.30)	<0.001
ALT (U/L), (median, IQR)	22.00 (17.00–30.00)	21.00 (17.00–28.00)	20.00 (15.00–26.00)	<0.001
AST (U/L), (median, IQR)	24.00 (20.00–28.00)	23.00 (19.00–28.00)	22.00 (19.00–27.00)	<0.001
Serum glucose (mg/dl), (median, IQR)	94.00 (87.00–106.00)	95.00 (87.00–108.00)	95.00 (88.00–109.00)	<0.001
Glycohemoglobin (%), (median, IQR)	5.60 (5.30–5.90)	5.60 (5.30–6.00)	5.70 (5.40–6.10)	<0.001
Uric acid (mg/dl), (median, IQR)	5.40 (4.50–6.40)	5.40 (4.50–6.50)	5.40 (4.50–6.50)	0.871
BUN (mg/dl), (median, IQR)	14.00 (12.00–18.00)	14.00 (11.00–18.00)	14.00 (11.00–17.00)	<0.001
Creatinine (umol/L), (median, IQR)	78.68 (61.90–89.28)	76.91 (61.90–90.17)	78.68 (63.65–92.82)	<0.001
ACR (mg/g), (median, IQR)	7.19 (4.50–14.71)	7.90 (4.89–17.25)	8.70 (5.26–19.38)	<0.001
eGFR (mL/min/1.73 m^2^), (median, IQR)	83.79 (70.51–99.42)	82.14 (68.28–98.20)	79.35 (65.62–94.01)	<0.001
Triglyceride (mg/dl), (median, IQR)	127.00 (86.00–193.00)	128.00 (88.00–192.00)	129.00 (90.00–189.00)	0.476
Cholesterol (mg/dl), (median, IQR)	199.00 (173.00–226.00)	200.00 (173.00–228.00)	199.00 (172.00–228.00)	0.071
BMI (kg/m^2^), (median, IQR)	27.81 (24.67–31.70)	28.45 (25.09–32.59)	28.79 (25.15–33.18)	<0.001
Hypertension (%)	42.07	45.50	49.63	<0.001
Coronary heart disease (%)	5.12	5.31	6.03	0.0369
Congestive heart failure (%)	3.33	4.80	5.39	<0.001
Stroke (%)	3.79	4.91	6.93	<0.001
Arthritis (%)	34.25	37.77	41.93	<0.001
Cancer (%)	12.77	12.67	14.32	0.0041
Diabetes (%)	20.21	23.56	25.35	<0.001
Albuminuria (%)	13.41	15.53	17.62	<0.001
Low-eGFR (%)	11.03	13.86	17.80	<0.001
CKD (%)	21.42	24.82	29.45	<0.001

Medians (IQR) for continuous variables: the *p*-value was calculated by the weighted linear regression model. (%) for categorical variables; the *p*-value was calculated by the weighted chi-square test. PIR, ratio of family income to poverty; AST, aspartate transaminase; ALT, alanine transaminase; BUN, blood urea nitrogen; BMI, body mass index; ACR, albumin: creatinine ratio; eGFR, estimated-glomerular filtration rate; CKD, chronic kidney disease.

**TABLE 2:** Baseline characteristics of study participants grouped by chronic kidney disease status.

	Overall	Non-chronic kidney disease	chronic kidney disease	*P*-value
	*N* = 23,175	*N* = 17,328	*N* = 5,847	
Age (year), (median, IQR)	60.00 (49.00–70.00)	57.00 (47.00–66.00)	70.00 (60.00–79.00)	<0.001
**Sex (%)**				<0.001
Male	49.82	48.15	54.75	
Female	50.18	51.85	45.25	
**Race (%)**				0.0212
Mexican American	5.79	6.02	4.91	
Other Hispanic	4.42	4.48	4.19	
Non-Hispanic White	75.06	74.80	76.04	
Non-Hispanic Black	9.28	9.17	9.69	
Other race	5.45	5.53	5.17	
**Education level (%)**				<0.001
Less than high school	6.86	6.00	10.20	
High school or GED	35.94	34.95	39.77	
Above high school	57.13	58.99	49.95	
Unknown	0.07	0.06	0.08	
**Smoking status (%)**				<0.001
Never	50.03	49.96	50.28	
Former	31.55	30.59	35.27	
Current	18.38	19.39	14.44	
Unknown	0.05	0.06	0.01	
PIR, (median, IQR)	2.33 (1.24–4.38)	2.51 (1.27–4.64)	1.94 (1.16–3.56)	<0.001
Serum iron (mg/dl), (median, IQR)	81.00 (62.00–104.00)	82.00 (63.00–105.00)	76.00 (58.00–98.00)	<0.001
Potassium (mmol/L), (median, IQR)	4.00 (3.80–4.29)	4.00 (3.80–4.20)	4.10 (3.84–4.40)	<0.001
Albumin (g/dl), (median, IQR)	4.20 (4.00–4.40)	4.20 (4.00–4.40)	4.10 (3.90–4.40)	<0.001
ALT (U/L), (median, IQR)	21.00 (16.00–28.00)	21.00 (17.00–29.00)	20.00 (15.00–26.00)	<0.001
AST (U/L), (median, IQR)	23.00 (19.00–28.00)	23.00 (19.00–28.00)	23.00 (19.00–28.00)	0.438
Serum glucose (mg/dl), (median, IQR)	95.00 (87.00–107.00)	94.00 (87.00–104.00)	100.00 (90.00–124.00)	<0.001
Glycohemoglobin (%), (median, IQR)	5.60 (5.30–6.00)	5.60 (5.30–5.90)	5.80 (5.40–6.50)	<0.001
Uric acid (mg/dl), (median, IQR)	5.40 (4.50–6.50)	5.30 (4.40–6.20)	6.00 (4.90–7.10)	<0.001
BUN (mg/dl), (median, IQR)	14.00 (11.00–18.00)	13.00 (11.00–16.00)	17.00 (13.00–23.00)	<0.001
Creatinine (umol/L), (median, IQR)	77.79 (62.76–90.17)	71.60 (61.88–84.86)	95.47 (76.02–114.92)	<0.001
ACR (mg/g), (median, IQR)	7.89 (4.87–16.98)	6.62 (4.47–10.69)	39.20 (10.61–96.63)	<0.001
eGFR (mL/min/1.73 m^2^), (median, IQR)	81.70 (68.14–97.34)	85.50 (74.25–100.00)	58.71 (49.15–81.19)	<0.001
Triglyceride (mg/dl), (median, IQR)	128.00 (88.00–191.00)	125.00 (86.00–187.00)	138.00 (96.00–206.00)	<0.001
Cholesterol (mg/dl), (median, IQR)	199.00 (173.00–227.00)	201.00 (175.00–228.00)	193.00 (165.00–224.00)	<0.001
BMI (kg/m^2^), (median, IQR)	28.32 (24.96–32.47)	28.20 (24.90–32.29)	28.75 (25.10–33.02)	<0.001
Dietary inflammatory index, (median, IQR)	1.68 (0.21–2.89)	1.58 (0.11–2.81)	1.98 (0.54–3.11)	<0.001
Hypertension (%)	45.73	39.31	64.79	<0.001
Coronary heart disease (%)	5.46	4.11	10.70	<0.001
Congestive heart failure (%)	4.5	2.56	10.26	<0.001
Stroke (%)	5.21	3.50	10.28	<0.001
Arthritis (%)	37.98	34.44	48.50	<0.001
Cancer (%)	13.21	11.42	20.14	<0.001
Diabetes (%)	23.04	17.76	38.67	<0.001

Medians (IQR) for continuous variables: the *p*-value was calculated by the weighted linear regression model. (%) for categorical variables; the *p*-value was calculated by the weighted chi-square test. PIR, ratio of family income to poverty; AST, aspartate transaminase; ALT, alanine transaminase; BUN, Blood Urea Nitrogen; BMI, body mass index; ACR, albumin: creatinine ratio; eGFR, estimated-glomerular filtration rate.

### 2.2 Study variables

In this study, we treated DII as an exposure variable and used two 24-h dietary reviews to obtain dietary information. There were 26 dietary parameters employed in calculating the DII, including carbohydrates, proteins, total fats, saturated fats, polyunsaturated fatty acid (PUFA), n-3 fatty acids, cholesterol, energy, alcohol, fiber, folate, iron, magnesium, zinc, selenium, monounsaturated fatty acid (MUFA), caffeine, niacin, riboflavin, thiamin, beta-carotene, Vitamins A/B6/B12/C/E. Initially, determine the mean intake level of each nutrient for the subjects, subtract the global average intake, and then divide the result by the standard deviation to compute the z-score. Subsequently, transform the z-scores into percentiles, and double the transformed values while subtracting “1” for data centering. The central percentile values for each food parameter are then multiplied by their respective inflammatory effect scores, resulting in a “food parameter-specific DII score.” Finally, each value was summed to obtain an individual “overall DII score” (10, 16).

The outcome variable was CKD, which was defined by albuminuria and/or low estimated glomerular filtration rate (low-eGFR) (17). Albumin-to-creatinine ratio (ACR) >30 mg/g was the diagnostic standard for albuminuria, and ACR was calculated using the urinary albumin/creatinine ratio. The diagnostic criteria for low-eGFR was eGFR < 60 ml/min/1.73 m^2^, and we calculated eGFR based on the Chronic Kidney Disease Epidemiology Collaborative equation (CKD-EPI Cr) (18).

Our study also incorporated a series of other factors as covariates, such as race, sex, age, educational level, smoking status, BMI, the poverty-to-income ratio (PIR), albumin, glucose, glycosylated hemoglobin, Alanine Aminotransferase (ALT), Aspartate Aminotransferase (AST), serum iron, potassium, blood urea nitrogen (BUN), uric acid, cholesterol, and triglycerides. Additionally, variations in health status for arthritis, cancer, hypertension, congestive heart failure, coronary heart disease, and diabetes were also enrolled as covariates.

### 2.3 Statistic analysis

In accordance with NHANES analytical guidelines, statistical analyses were conducted with appropriate sampling weights. Continuous data were given as medians [interquartile range (IQR)] and categorical variables as percentages. The effect of DII on three outcome variables, including CKD, albuminuria, and low-eGFR, was analyzed under three different models using multiple logistic regression. Model 1 did not incorporate any adjustment for covariates. Model 2 incorporated race, age, and sex. Model 3 incorporated more than just the three variables mentioned above, but also education level, smoking status, PIR, BMI, albumin, glucose, glycosylated hemoglobin, ALT, AST, serum iron, potassium, cholesterol, triglycerides, BUN, uric acid, hypertension, coronary heart disease, congestive heart failure, stroke, arthritis, cancer, and diabetes. Furthermore, smoothed curve fitting and threshold effect evaluation were used to evaluate the non-linear connection of DII with these three outcome variables. Subgroup analyses were carried out to further examine the correlation of DII with these outcome variables. *P* < 0.05 has been identified to be statistically significant in this study. R (version 4.2.3) and EmpowerStats (version 2.0) were used for statistical calculations and graphics.

## 3 Results

### 3.1 Baseline characteristics

This research included 23,175 individuals, with a mean age of 60. A total of 49.82% of them were men, and 50.18% were women. After treating the DII as a quantile variable, the basic information for all subjects was presented in Table 1. According to our statistics, those with greater DII tertiles were more likely to have hypertension, coronary heart disease, congestive heart failure, arthritis, cancer, stroke, diabetes, and CKD than people in other categories.

There were 5,847 persons with CKD among all participants, accounting for 25.23% of the total. Basic information on subjects grouped by CKD situation was shown in Table 2. The findings suggested that CKD was more prevalent in older, less educated, lower PIR, male, and non-Hispanic white groups. Those with CKD possessed higher levels of potassium, glucose, glycosylated hemoglobin, uric acid, BUN, triglycerides, BMI, and DII. In addition, these people had higher rates of hypertension, arthritis, cancer, stroke, congestive heart failure, coronary heart disease, and diabetes than non-CKD people. In contrast, serum iron, albumin, ALT, and cholesterol levels were lower in the CKD population.

### 3.2 Association between DII and CKD, low-eGFR, and albuminuria

The results of the multiple regression analysis were shown in Table 3, including the association of DII with CKD, low-eGFR, and albuminuria. Our results indicated that higher DII scores were connected with a higher risk of CKD. This relation was significant in all models (Model 1, OR = 1.11, 95% CI: 1.09, 1.13, *p* < 0.0001; Model 2, OR = 1.10, 95% CI: 1.08, 1.12, *p* < 0.0001; Model 3, OR = 1.08, 95% CI: 1.05, 1.10, *p* < 0.0001). In the unadjusted model (model 1), each one-unit increase in DII score increased the risk of CKD in subjects by 11%, whereas, in the fully adjusted model (model 3), this risk increased by 8%. After transforming DII into tertiles, this relation still maintained its statistical significance. In the unadjusted model, individuals in the highest tertile of DII scores had a significantly increased risk of CKD of 53% (OR = 1.53, 95% CI: 1.42, 1.65; p for trend <0.0001) compared with participants in the bottom tertile of DII scores, and this risk increased by 35% (OR = 1.35, 95% CI: 1.23, 1.48; p for trend <0.0001) after adjusting for all covariates.

**TABLE 3:** Association between the dietary inflammatory index and CKD, low-eGFR and albuminuria.

Exposure	Model 1 [OR (95% CI)], *P-*value	Model 2 [OR (95% CI)], *P-*value	Model 3 [OR (95% CI)], *P-*value
**CKD**			
DII (continuous)	1.11 (1.09, 1.13) <0.0001	1.10 (1.08, 1.12) <0.0001	1.08 (1.05, 1.10) <0.0001
**DII (categories)**			
Tertile 1	Reference	Reference	Reference
Tertile 2	1.21 (1.12, 1.30) <0.0001	1.20 (1.11, 1.30) <0.0001	1.12 (1.02, 1.23) 0.0175
Tertile 3	1.53 (1.42, 1.65) <0.0001	1.48 (1.36, 1.60) <0.0001	1.35 (1.23, 1.48) <0.0001
P for trend	<0.0001	<0.0001	<0.0001
**Low-eGFR**			
DII (continuous)	1.14 (1.12, 1.17) <0.0001	1.15 (1.12, 1.18) <0.0001	1.16 (1.13, 1.19) <0.0001
**DII (categories)**			
Tertile 1	Reference	Reference	Reference
Tertile 2	1.30 (1.18, 1.43) <0.0001	1.34 (1.20, 1.48) <0.0001	1.29 (1.13, 1.47) 0.0001
Tertile 3	1.75 (1.59, 1.92) <0.0001	1.78 (1.61, 1.97) <0.0001	1.85 (1.62, 2.10) <0.0001
P for trend	<0.0001	<0.0001	<0.0001
**Albuminuria**			
DII (continuous)	1.09 (1.06, 1.11) <0.0001	1.07 (1.05, 1.09) <0.0001	1.02 (1.00, 1.05) 0.0742
**DII (categories)**			
Tertile 1	Reference	Reference	Reference
Tertile 2	1.19 (1.09, 1.30) 0.0002	1.15 (1.05, 1.26) 0.0033	1.04 (0.94, 1.16) 0.4332
Tertile 3	1.38 (1.26, 1.51) <0.0001	1.27 (1.16, 1.39) <0.0001	1.06 (0.96, 1.18) 0.2384
P for trend	<0.0001	<0.0001	0.2368

In sensitivity analysis, the dietary inflammatory index was converted from a continuous variable to a categorical variable (tertiles). 95% CI: 95% confidence interval. OR, odd ratio. Model 1: No covariates were adjusted. Model 2: adjusted for sex, age, and race. Model 3: adjusted for sex, age, race, education level, smoking status, PIR, BMI, albumin, serum glucose, glycohemoglobin, ALT, AST, serum iron, potassium, cholesterol, triglycerides, BUN, uric acid, hypertension, coronary heart disease, congestive heart failure, stroke, arthritis, cancer, and diabetes.

A similar positive correlation was also observed for DII with low-eGFR. We noted a significant correlation between DII and low-eGFR in all models (Model 1, OR = 1.14, 95% CI: 1.12, 1.17, *p* < 0.0001; Model 2, OR = 1.15, 95% CI: 1.12, 1.18, *p* < 0.0001; Model 3, OR = 1.16, 95% CI: 1.13, 1.19, *p* < 0.0001). Without adjusting for any covariates (model 1), the risk of low-eGFR in subjects increased by 14% for each unit increase in DII, whereas this effect value further increased to 16% after adjusting for all covariates (model 3). This positive association persisted when DII was transformed into tertiles, too. Individuals in the highest tertile of DII were 75% more likely to have a low-eGFR than individuals in the lowest tertile in the unadjusted model (OR = 1.75, 95% CI: 1.59, 1.92; p for trend <0.0001), whereas in the fully adjusted model, this likelihood was 85% higher (OR = 1.85, 95% CI: 1.62, 2.10; p for trend <0.0001).

Our results showed a positive correlation between DII and albuminuria in all models, however, this correlation was not significant in the fully adjusted model (Table 3). The likelihood of albuminuria increased by 9% for each unit increase in DII in the unadjusted model (OR = 1.09, 95% CI: 1.06, 1.11, *p* < 0.0001) and by 7% in the minimally adjusted model (OR = 1.07, 95% CI: 1.05, 1.09, *p* < 0.0001). Both of these results were statistically significant. This correlation remained significant even when DII was converted to a tertile variable. However, the relation of DII to albuminuria was not statistically significant after adjusting for all covariates, regardless of whether DII was a continuous variable or a categorical variable.

**FIGURE 2:**
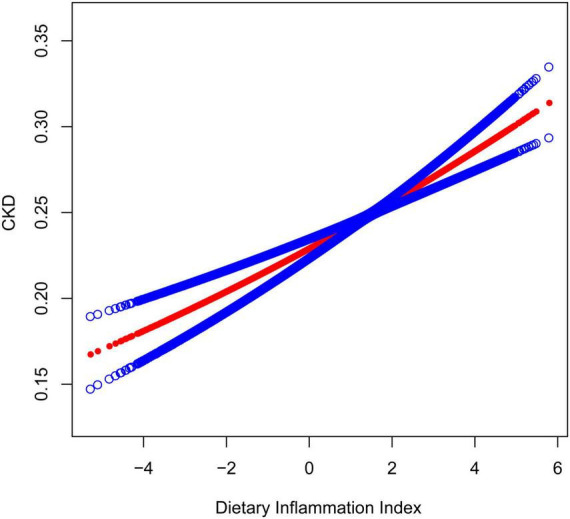
The association between dietary inflammatory index and CKD. The solid red line represents the smooth curve fit between variables. Blue bands represent the 95% confidence interval from the fit. The model adjusted for sex, age, race, education level, smoking status, PIR, BMI, albumin, glucose, glycosylated hemoglobin, ALT, AST, serum iron, potassium, cholesterol, triglycerides, BUN, uric acid, hypertension, coronary heart disease, congestive heart failure, stroke, arthritis, cancer, and diabetes.

**FIGURE 3:**
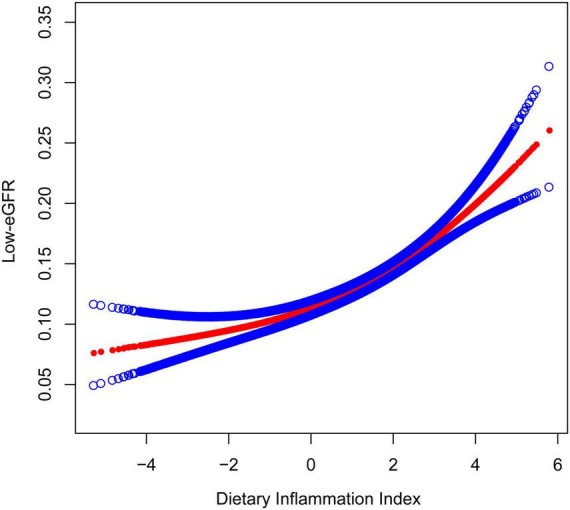
The Association between dietary inflammatory index and low-eGFR. The model adjusted for sex, age, race, education level, smoking status, PIR, BMI, albumin, glucose, glycosylated hemoglobin, ALT, AST, serum iron, potassium, cholesterol, triglycerides, BUN, uric acid, hypertension, coronary heart disease, congestive heart failure, stroke, arthritis, cancer, and diabetes.

Figures 2, 3 depicted the non-linear relationships in the fully adjusted model between DII and CKD, as well as between DII and low-eGFR, through smooth curve fitting. It was worth noting that we found a U-shaped relation between DII and low-eGFR with a turning point of 1.6 (Table 4).

**TABLE 4:** Threshold effect analysis of dietary inflammatory index on low-eGFR using two-piecewise linear regression model.

Exposure	Adjusted OR (95% CI), *P*-value
Fitting by the standard linear model	1.16 (1.13, 1.19) <0.0001
Fitting by the two-piecewise linear model	
Inflection point	1.6
DII < 1.6	1.10 (1.04, 1.16) 0.0004
DII > 1.6	1.25 (1.17, 1.34) <0.0001
Log likelihood ratio test	0.014

The model adjusted for sex, age, race, education level, smoking status, PIR, BMI, albumin, glucose, glycosylated hemoglobin, ALT, AST, serum iron, potassium, cholesterol, triglycerides, BUN, uric acid, hypertension, coronary heart disease, congestive heart failure, stroke, arthritis, cancer, and diabetes.

### 3.3 Subgroup analysis

For the associations of DII with CKD, DII with low-eGFR, and DII with albuminuria, we performed subgroup analyses including age, sex, BMI, smoking status, diabetes status, and hypertension status in the completely adjusted model (Table 5). Results indicated that the positive associations of DII with CKD and DII with low-eGFR remained stable and were not significantly different in all subgroups (all P for interaction >0.05). Consistent with prior results, we did not observe any statistically significant correlation between DII and albuminuria in all subgroups in the fully adjusted model, except in the non-hypertensive population (OR = 1.06, 95% CI: 1.02, 1.10). This group difference based on having or not having hypertension was statistically significant (P for interaction = 0.0095).

**TABLE 5:** Association between dietary inflammatory index and CKD, low e-GFR, and albuminuria in different subgroups.

	CKD		Low-eGFR		Albuminuria	
	OR (95% CI) *P*-value	*P* for interaction	OR (95% CI) *P*-value	*P* for interaction	OR (95% CI) *P*-value	*P* for interaction
**Age**		0.9774		0.7612		0.8729
40∼59	1.08 (1.04, 1.12) <0.0001		1.17 (1.09, 1.24) <0.0001		1.03 (0.99, 1.07) 0.1865	
Age ≥ 60	1.08 (1.05, 1.10) <0.0001		1.15 (1.12, 1.19) <0.0001		1.02 (0.99, 1.05) 0.1120	
**Sex**		0.8213		0.2081		0.4738
Male	1.08 (1.05, 1.11) <0.0001		1.18 (1.13, 1.23) <0.0001		1.01 (0.98, 1.05) 0.4123	
Female	1.07 (1.04, 1.11) <0.0001		1.14 (1.09, 1.19) <0.0001		1.03 (1.00, 1.07) 0.0780	
**Smoking status**		0.7299		0.2482		0.6440
Never	1.07 (1.04, 1.10) <0.0001		1.14 (1.09, 1.18) <0.0001		1.02 (0.98, 1.05) 0.3548	
Former	1.09 (1.05, 1.13) <0.0001		1.16 (1.11, 1.22) <0.0001		1.04 (0.99, 1.08) 0.0864	
Current	1.07 (1.01, 1.13) 0.0156		1.27 (1.15, 1.40) <0.0001		1.00 (0.95, 1.07) 0.8721	
**BMI**		0.1005		0.8468		0.6289
Normal weight	1.09 (1.05, 1.14) <0.0001		1.17 (1.10, 1.24) < 0.0001		1.04 (0.99, 1.09) 0.1081	
Overweight	1.09 (1.05, 1.13) <0.0001		1.17 (1.11, 1.23) <0.0001		1.03 (0.99, 1.07) 0.1988	
Obese	1.05 (1.02, 1.09) 0.0029		1.15 (1.10, 1.21) <0.0001		1.01 (0.97, 1.04) 0.7450	
**Hypertension**		0.1972		0.1266		0.0095
Yes	1.06 (1.03, 1.09) <0.0001		1.13 (1.08, 1.18) <0.0001		1.00 (0.97, 1.03) 0.8182	
No	1.09 (1.06, 1.13) <0.0001		1.18 (1.14, 1.23) <0.0001		1.06 (1.02, 1.10) 0.0019	
**Diabetes**		0.7534		0.8810		0.8788
Yes	1.07 (1.03, 1.11) 0.0005		1.16 (1.12, 1.20) <0.0001		1.02 (0.99, 1.05) 0.1773	
No	1.08 (1.05, 1.11) <0.0001		1.16 (1.10, 1.23) <0.0001		1.02 (0.99, 1.07) 0.2268	

In subgroup analyses of CKD, low-eGFR, and albuminuria stratified by age, sex, smoking status, BMI, hypertension status, and diabetes status, the model adjusted for sex, age, race, education level, smoking status, PIR, BMI, albumin, glucose, glycosylated hemoglobin, ALT, AST, serum iron, potassium, cholesterol, triglycerides, BUN, uric acid, hypertension, coronary heart disease, congestive heart failure, stroke, arthritis, cancer, and diabetes. However, the model was not adjusted for the stratification variables themselves.

## 4 Discussion

In this research involving 23,175 participants, we assessed the relation between DII and CKD in individuals aged 40 years and older in the United States. We found that higher levels of DII were significantly and positively correlated with a higher risk of CKD and low-eGFR in these middle-aged and older adults. This association remained stable across strata of age, sex, BMI, smoking status, diabetes mellitus status, and hypertension status, and no significant stratification differences were seen. In addition, there was a U-shaped relation between DII and low-eGFR with a turning point of 1.6.

The link of diet-induced inflammation to prevalent chronic illnesses has been shown in an array of earlier research. Shate Xiang et al. discovered that DII was positively linked to rheumatoid arthritis risk and superimposed on other risk variables (19). Through meta-analysis, some researchers found that a greater DII score might raise the risk of colorectal cancer and was strongly associated with an elevated cancer risk of the ovaries among postmenopausal females (20, 21). It is essential to note that the greater the positive DII score, the greater the pro-inflammatory nature of the diet. Therefore, adhering to a balanced and anti-inflammatory diet may potentially lower the risk of cancer development. An increasing corpus of research has indicated that diet has a significant impact on the emergence of cardiovascular disease (CVD) (11). A cross-sectional study involving 48,733 participants indicated that a rise in DII could heighten the danger of CVD among individuals aged 18 and above in the United States. Furthermore, this occurrence showed a more significant effect on women. At the same time, there was continuing evidence of a link between DII and mortality in the population. For instance, it has been shown that DII has a positive association with the prevalence of diabetes and a diet with high levels of pro-inflammatory compounds could potentially contribute to higher mortality rates among people with diabetes (22).

In the past few years, numerous investigations have demonstrated there were certain links between DII and CKD. In a cross-sectional investigation, Ying Huang and colleagues discovered that a rise in DII might be contributory to the development of sarcopenia in individuals with CKD and that an anti-inflammatory diet might be protective against CKD-associated sarcopenia (23). It has also been suggested that treatments using an anti-inflammatory diet might lower rates to get hyperparathyroidism in patients with CKD (24). Moreover, Ying Huang et al. found that the association of elevated energy-adjusted dietary inflammatory index (E-DII) with increased risk of 5-year all-cause and cardiovascular mortality in individuals with CKD was not modified by other factors, while subgroup analyses showed that individuals with worsening renal function were more vulnerable to the effects of E-DII (25). With the aging of populations worldwide, researchers are increasingly concentrating on middle-aged and older populations, including DII-related studies. As it is widely acknowledged, there exists an anti-aging protein in plasma, soluble Klotho (S-Klotho). A research had indicated a negative association between S-Klotho and pro-inflammatory food patterns evaluated based on DII in people over 40 years old (26). Furthermore, these middle-aged and older people with high DII scores also had an increased likelihood of suffering from low HDL cholesterol, hyperglycemia, and metabolic syndrome (27). Epidemiologic studies of DII and CKD are emerging. Zeng’s team found that higher DII scores were independently associated with increased odds of CKD stages 3–5 in adults aged 50 years and older. And its linear association remained stable across stratified analyses of age, gender, and ethnicity (28). This is similar to our findings. And in a large retrospective cohort study with a mean follow-up of 132.03 months, Yan et al. observed an association between DII and the risk of all-cause mortality in a population of US adults with CKD. The results showed that the risk of death was more pronounced in those with higher DII scores, especially in those under 65 years of age (29). However, further research is needed to delve deeper into the relationship between DII and CKD.

There have been many studies on DII and CKD, but the exact mechanism was still unknown. Below are some potential pathological processes that could exist. Inflammation plays a key role in CKD progression, and poor dietary habits can contribute to this inflammatory response, impacting kidney health through diverse mechanisms. High-calorie and high-fat diets can lead to obesity, and the chronic low-grade inflammation that it triggers is one of the common initiators of CKD. Adipose tissue releases lipid mediators and cytokines such as TNF-α and IL-6, which can trigger systemic inflammation and cause direct damage to the kidneys (30). The hyperglycemic state resulting from a prolonged high-sugar diet triggers the production of glycosylation end-products (AGEs), which activate inflammatory pathways and lead to insulin resistance, further contributing to the development of inflammation (31). Micronutrients such as zinc, selenium and antioxidants such as vitamins C and E are essential for maintaining the health of living organisms (32). Inadequate diets may lead to deficiencies in these nutrients, potentially making the kidneys more susceptible to damage from oxidative stress, which can trigger an inflammatory response. Chronic high-salt diets can alter the structure and function of small renal arteries. In addition, it has been shown that high salt intake independently promotes immune activation of macrophages, exacerbating inflammation and accompanying renal deterioration (33). Previous studies have shown that there is an interaction between chronic inflammation and CKD (34). For one thing, chronic systemic inflammation could cause renal failure and speed up the development of renal fibrosis and CKD (35, 36). And for another, the uremic setting in the advanced stage of CKD would lead to uremic inflammation, which is a long-term, low-grade inflammation related to aging (37–39). Plenty of cytokines and inflammatory indicators, like TNF-α, CRP, IL-1, and IL-6, have been related to the development of CKD (40). These inflammatory factors are closely linked to the triggering of the nuclear factor-κB (NF-κB) cascade (41). Such a pathway would induce mitochondrial dysfunction and increased amounts of reactive oxygen species (ROS) (42, 43), and NF-κB may cause an inflammatory cytokine feedback loop that might maintain kidney inflammation (44). Thus, a more pro-inflammatory diet may enhance the expression of inflammatory factors, thereby promoting an inflammatory state in the body, leading to an increased risk of CKD.

This is the first study we know of to look at the relation of DII to CKD in individuals aged 40 years and older. Second, the participants in this study were selected from the national population through a stratified, multistage sampling procedure and were well represented, so our outcomes could apply to the whole group of middle-aged and older adults in this country. Most notably, the large nationwide sample size made it possible to perform subgroup analyses by age, sex, diabetes status, and hypertension status. The results revealed that the positive relation of DII to CKD remained consistent in all subgroups. However it is necessary to be aware of the shortcomings of our study. In the first instance, because of the cross-sectional nature of this study, we could not establish a clear causal connection of DII with CKD. Secondly, it is generally recognized that two consecutive 24-h dietary recall interviews might only be used as a reference, and there might be a degree of memory uncertainty that could not accurately reflect long-term dietary habits. Thirdly, whether or not to perform hemodialysis is crucial for the study of CKD patients, but we did not include it as a variable in our study due to the lack of relevant data in the NHANES database. Fourth, disparities exist among CKD patients at various stages, necessitating more comprehensive investigations in the future to ascertain the generalizability of our findings. Finally, we only used the American population as the research sample, and the research results cannot represent all populations. Further research is needed to demonstrate our findings in the future.

## 5 Conclusion

As a result, our findings have demonstrated that increased DII was substantially and positively correlated with elevated hazard of CKD in individuals of middle and older age. This highlighted the importance of an anti-inflammatory diet in reducing the risk of CKD in this group. But further extensive prospective studies are needed to test the validity of our findings.

## Data availability statement

The original contributions presented in the study are included in the article/supplementary material, further inquiries can be directed to the corresponding author.

## Ethics statement

The studies involving humans were approved by the National Center for Health Statistics Research Ethics Review Board. The studies were conducted in accordance with the local legislation and institutional requirements. The participants provided their written informed consent to participate in this study. Written informed consent was obtained from the individual(s) for the publication of any potentially identifiable images or data included in this article.

## Author contributions

MG: Conceptualization, Data curation, Investigation, Methodology, Software, Writing – original draft, Writing – review and editing. YL: Data curation, Writing – original draft. XuL: Data curation, Writing – original draft. XiL: Writing – review and editing. YX: Writing – review and editing. DZ: Conceptualization, Writing – review and editing.

## Footnotes

1
www.cdc.gov/nchs/nhanes/
